# Thoughts Suggested by the Death of Richard Wagner

**Published:** 1883-03

**Authors:** James I. Tucker

**Affiliations:** Chicago


### ARTICLE II.

### THOUGHTS SUGGESTED BY THE DEATH OF RICHARD WAGNER.

By DR. JAMES I. TUCKER, Chicago.

On the second étage of the Palais Vendremint in the city of Venice, on the 14th inst., Richard Wagner, the great composer, died at the age of seventy. He had long been a sufferer from cardiac disease, the exact nature of which the dispatches do not state. On Tuesday he ordered his gondola for 3 o'clock, intending to take his customary promenade on the Grand Canal. As he was about to leave he was attacked by "a fit of asthma." He murmured, "I feel very bad," became unconscious, and at 4 o'clock died, surrounded by his wife, his four daughters and his favorite son, Siegfried. Thus ended the eventful career of him, who by genius, application, resolution and perseverance had become doubtless the greatest composer that ever lived; greatest not in the intensity and originality of his genius, but in that he took up the lyric drama at the zenith of its evolution and carried it forward to a point so much higher that centuries will probably elapse before a greater than he, in the light of future science, will venture to undertake a similar task; when what is now the

Music of the Future shall have become a thing of the past. The peer of Beethoven he had the massive Beethoven foundation as an inheritance upon which to erect his colossal superstructure. The great movement inaugurated by Beethoven expanded into the Wagnerian opera, a complete lyric structure in which the correlation of the Fine Arts was recognized and made the corner-stone, a structure in which there was no disproportion or discord, but wherein music, poetry, painting, action and oratory were one. "There is poetry," wrote Goethe, "there is painting, there is music and song, and there is the wit of representation. When all these arts and charms of youth and beauty shall work together upon the same evening and at the same degree of excellence, there will be a feast with which no other can be compared." Wagner accomplished this, and herein lies his greatness. But he could not be simply a musician, or a painter, or an actor, or an orator; he had to be all in one, and had he not lived when Science had pushed itself forward to the foreground he could not have achieved what he has. Like the physician, he pressed all the collateral science into service. It was necessary to understand the law of the correlation and conservation of forces, and of vibratory motion, together with the laws of light and sound. He had to drink deep of the scientific nectar and feed on the scientific ambrosia, and be fully swayed by the scientific bias.

He lived at a time when society, government, the church and medicine had been modified by science. He saw, too, that there are many thoughts and feelings which music can, but which language is wholly inadaquate to express. With music conceived as simply a tickler of the emotions, he had no sympathy; it was only after he had studied the exact science of counterpoint, that he recognized in music a field for the highest intellectual exerercise. The days of emotional music, as well as emotional preaching, are fast dying away, and soon physicians will cease to classify music as basilar, but perceive that it is in the highest degree cerebral—cease to associate it with the passions, but perceive in ~~it~~ the light of the most exalted reason. If it had not been for the scientific bias, Shakspere and Gœthe and Lessing could never have written; without it Wagner could never have composed. In the choice of themes, too, Wagner was great, for he selected the classic myths in which he perceived the deep significance underlying fundamental thoughts constituting a law of the human mind. But while Wagner clearly perceived the correllation of the fine arts, and that harmony was the connecting link between them, it is doubtful whether he knew enough of the human organism to include medicine among them. It is possible that he was so much occupied with the branches of fine art, which had a special bearing on the development of the lyric drama that even in his criticisms, he could give no attention to medicine as a fine art, though from the quality of his mind we would be led to suppose that he would be among the first who would be able to comprehend a truth so evident to the physician. But when we consider how other artists of eminence have failed to recognize the relation of medicine to the fine arts, it may be that Wagner also failed to recognize it, and after all, it would not be surprising when we think that until very recently the harmony of the human body was ill-understood, because our knowledge of the mechanism of the nervous system has but just emerged from its infancy. Heinrich Rolfhs, however, does not hesitate to sharply criticise August von Eye for having failed to include medicine among the fine arts; and he is right, for it is evident to the more advanced medical mind, which perceives the law of harmony operating in the human organism, and that therapeutics has but a single aim and that to restore harmony when disturbed, that medicine is not only one of the fine arts, but the finest among them, because it has to do with human life.

